# Gut microbiota in anxiety and depression: Pathogenesis and therapeutics

**DOI:** 10.3389/fgstr.2022.1019578

**Published:** 2022-10-12

**Authors:** Stefano Bibbò, Salvatore Fusco, Gianluca Ianiro, Carlo Romano Settanni, Daniele Ferrarese, Claudio Grassi, Giovanni Cammarota, Antonio Gasbarrini

**Affiliations:** ^1^ CEMAD Digestive Disease Center, Fondazione Policlinico Universitario A. Gemelli IRCCS - Università Cattolica del Sacro Cuore, Rome, Italy; ^2^ Departement of Neuroscience, Università Cattolica del Sacro Cuore - Fondazione Policlinico Universitario A. Gemelli IRCCS, Rome, Italy

**Keywords:** FMT, gut-brain axis, probiotic, prebiotics, antibiotic, synbiotic

## Abstract

Depression and anxiety disorders represent a burdensome clinical issue. Considering the unsatisfactory clinical response of some patients to antidepressant therapy, new personalized approaches are being studied. In recent years, pre-clinical and clinical studies have investigated the role of intestinal microbiota demonstrating the importance of the gut-brain axis in these diseases. Indeed, gut microbes are able to interact with the brain interfering with behavior through some mechanisms such as amino acid metabolism, short–chain fatty acids, vagus nerve, endocrine signaling and immune responses. Experiments of gut microbiota transfer from subjects with major depression to animal models corroborated the causative role of intestinal microbes in mood disorders and anxiety. Furthermore, the incidence of dysbiosis in patients with anxiety and depression suggests a potential role for gut microbiota modulators in the treatment of these disorders. In particular, several probiotics and synbiotics have been shown to be effective in improving clinical symptoms, promising results have emerged also from fecal microbiota transplantation, but the evidence is still limited. These promising results switch on the use of gut microbiota modulators as an adjunctive tool to anti-depressant therapy. Developing pharmaceutical or nutraceutical strategies to modify the composition of gut microbiota may offer novel and personalized therapeutic tools against anxiety and depression.

## Introduction

Anxiety and mood disorders represent an alarming clinical issue, as well as cause of disability and mortality worldwide (1). Unfortunately, the mechanisms triggering these diseases have not yet been fully understood. Several factors such as oxidative stress (2), impaired signaling by neurotrophic factor (3) or chronic inflammation (4) have been hypothesized to be involved in the development and susceptibility of mood disorders, which presumably are caused by an interplay between genetics and environmental factors (5, 6). To date, the lack of this knowledge has a negative effect on the efficacy of common therapies, so there is a need for personalized treatment for these patients (7). In this regard, considering the pathophysiological role of the intestinal microbiome, the development of innovative therapies for these disorders can be hypothesized. Gut microbes are able to produce most neurotransmitters, influencing neurochemistry and behavior *via* the so-called “gut-brain axis” (8). Moreover, the high prevalence of stress-related psychiatric symptoms in patients with gastrointestinal disorders supports the link between gut microbiota changes and psychiatric disorders (9). The functional crosstalk among enteric microorganisms, gut and brain may occur through multiple mechanisms, including metabolic and neuroimmunological pathways. Finally, developing pharmaceutical or nutraceutical strategies to modify the composition of gut microbiota may offer novel and personalized therapeutic tools against anxiety and depression, which we will discuss below.

## Gut microbiota regulates anxiety-like and depression-like behavior: Evidences from animal studies

Despite the limitations represented mainly by the difference in the composition of the human and murine microbiota, and the difficulty of translate the findings from experimental models to patients where no complete ablation of the microbiota can be achieved, studies on rodents indicate that gut microbiota influences brain function and may impact on the behavior (10). Experimental approaches used to study the microbiota-gut-brain axis included the treatment with probiotics/antibiotics, the induction of gut inflammation by injection of enteric bacterial pathogens, the use of germ-free (GF)/gnotobiotic animals and the human diseases-related fecal microbiota transplantation (FMT) (11). The main advantages of studies performed on murine experimental models are the efficacy of behavioral tests to reveal changes similar to what observed in patients affected by anxiety or depression (12), and the possibility to analyze the effects of a single bacterial phylum or species on behavior. Animal studies suggested that changes in the microbiota induced brain modifications at both molecular and behavioral level. Mice treated with a cocktail of non-absorbable antibiotics showed changes of intestinal microbiota profile (i.e., a reduction of *Shigella*, *Bacteroides* and *Klebsiella* genera and an increase of *Actinobacter* and *Lactobacillus* populations) in parallel with greater exploratory activity (13). This anxiolytic effect was accompanied by an increase of brain-derived neurotrophic factor (BDNF) levels in the hippocampus and amygdala. More importantly, the authors did not observe the same responses in animals intraperitoneally injected with the antibiotics or in germ-free mice to which the drugs were administered by gavage. Moreover, gut microbiota seems to be involved in the diet-induced brain modification. High fat diet (HFD) is a well-established experimental model able to induce changes of both insulin and leptin signaling into the brain, anxiety and memory deficits (14–16). *Soto* and colleagues demonstrated that in HFD-fed mice, oral treatment with antibiotics modified the levels of neuromodulators such as tryptophan, γ-aminobutyric acid (GABA) and BDNF, ameliorated brain insulin signaling and counteracted anxiety and depression (17). In addition, the authors documented that these effects were transferable to germ-free mice by FMT.

Indeed, a large part of these studies based on the transferability of behavioral traits from donor mice to germ-free animals *via* the intestinal microbiota. For instance, BALB/c mice have anxiety-like behavior but, when they were colonized with the microbiota from Swiss mice, they acquired a more exploratory behavior. Accordingly, germ-free Swiss mice colonized with the intestinal bacteria from BALB/c mice exhibited a more anxious behavior (13). More recently, it has been showed that mice transplanted with fecal microbiota from Irritable bowel syndrome (IBS) patients exhibited intestinal barrier dysfunction, immunological activation, and anxiety-like behavior (18). More generally, intestinal microbiota appears to influence the stress response of rodents. *Sudo* and colleagues demonstrated that plasma levels of both ACTH and corticosterone were more prone to increase upon restraint stress in GF mice than in microbiota-competent animals (19). Moreover, the colonization by *Bifidobacterium infantis* of germ free mice was able to fully reverse these effects, revealing a causative role for the gut microbiota in modulating stress responses. Accordingly, the reduced expression of inflammatory interleukins and increased the amount of BDNF in the hippocampus was obtained by oral intake of Bifidobacterium, causing anxiolytic and antidepressant effects in mice (20). Bifidobacterium administration has been also shown to offer resilience to chronic social defeat stress in mice (21). In addition, three independent studies found altered concentrations of neurotransmitters and neurotrophic factors in the brain, and reduced anxiety in GF mice (22–24). These neurochemical and behavioral findings are not actually in agreement, because enhanced hypothalamic–pituitary–adrenal (HPA) axis is usually related to increased anxiety-like behavior. *Clarke* and colleagues also reported elevated concentrations of tryptophan, the precursor of serotonin, in the plasma and a significant increase of serotonin metabolites in the hippocampus of male GF mice compared with control animals (24). Serotonin is an excitatory neurotransmitter produced also in the gut and able to counteract anxiety and depression at central level (25). Metabolomics studies revealed elevated serum tryptophan and less serum serotonin in GF mice compared to controls (26). However, whether changes in serotonin and neurotrophic factors (e.g., BDNF) are involved in the gut microbiota-dependent modification of anxiety-like behavior remains to be elucidated.

**Figure 1:**
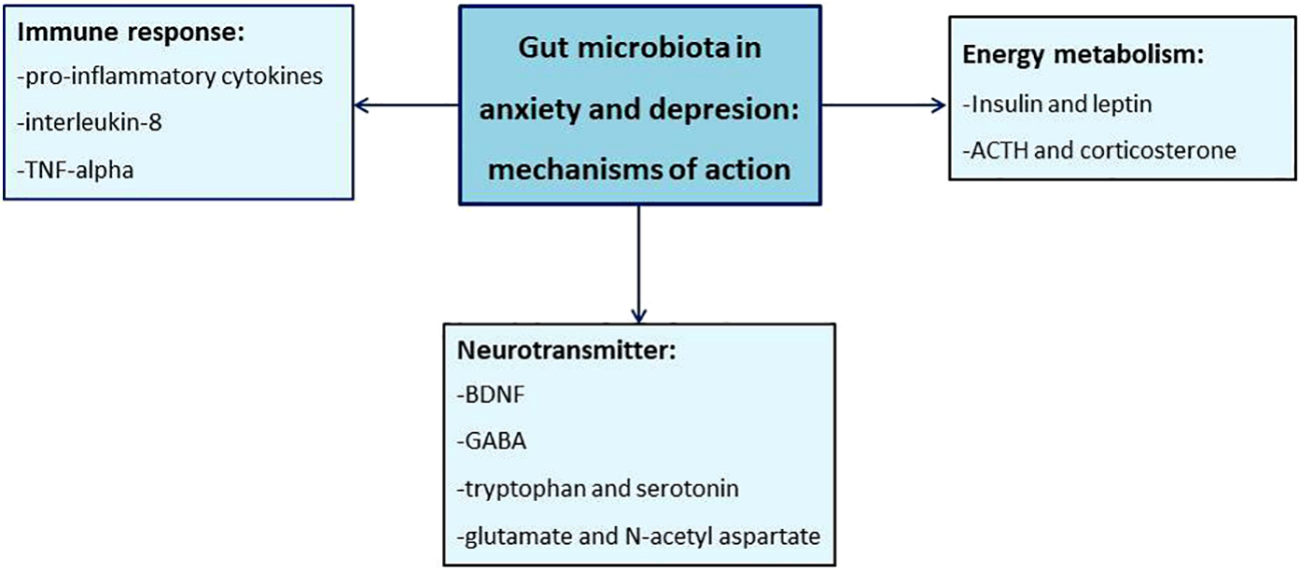
Role of the gut microbiota in the development of mood disorders. Some pathophysiological mechanisms underlying the development of anxiety and depression have been proposed, in particular the balance of immunological, neurotransmitter and hormonal mechanisms is the proposed orchestror.

Rodent models have provided the mechanisms by which the gut microbiota may modulate depression-like behaviors. Maternal separation is a model of early life stress that induces anxiety and depression by altering HPA axis, immune system and aminoacid metabolism along with affecting microbiota composition (27, 28). More recently, *De Palma* and colleagues demonstrated that maternal separation of GF mice did not induce depressive or anxiety behavior despite it caused increase of circulating corticosterone (29). This study suggests that gut microbiota is not required for stress-induced changes in HPA axis activity but it is necessary for development of anxiety and depression-like behaviors. Therefore, intestinal microbes appeared to regulate stress responses in the brain of animal models and this evidence stimulated the possibility of using probiotic treatments to modulate brain function in physiological and pathological conditions (30). A plethora of probiotic agents have been tested in rodent models of anxiety and depression. *Bifidobacterium* and *Lactobacillus* are the main genera that have provided beneficial effects on neurological disorders (31). *Bifidobacterium infantis* has been shown to have antidepressant effect promoting antidepressant-like performance in the forced swim test, a widely used test to evaluate the efficacy of antidepressant drugs (32). Supplementation of *Bifidobacterium infantis* also counteracted the maternal separation-induced increase of both plasma tryptophan and pro-inflammatory cytokines, which have been demonstrated to play a role in the pathophysiology of depression (33). Many studies also clarified the mechanisms underlying the effects of probiotics on brain functions. Several studies focalized the attention on the ability of probiotics to modulate the inflammatory response of the organism. *Lactobacillus rhamnosus* has been proved to inhibit *in vitro* the *Salmonella* enterica-related synthesis of pro-inflammatory interleukin-8 and tumor necrosis factor alpha (34). This bacterial strain has been also found to induce region-dependent changes in GABA receptor expression in the brain. More importantly, *Lactobacillus rhamnosus* administration reduced in mice the stress-dependent increase of corticosterone levels and counteracted the related anxiety- and depression-like behavior. Moreover, the beneficial effects of this probiotics were abolished in vagotomized animals (35). More recently, *Janik* and colleagues documented by magnetic resonance spectroscopy that chronic treatment with *Lactobacillus rhamnosus* induced significant changes in the concentration of neurotransmitters such as glutamate, N-acetyl aspartate, and GABA into the brain (36). It suggests that probiotics could affect brain activity by regulating neurochemical pathways underlying synaptic transmission and plasticity. In addition, administration of *Bifidobacterium Infantis* enhanced the expression of BDNF and N-methyl-D-aspartate receptor subunit 2a, which are molecules involved in learning and memory (19). Collectively, these studies prompt the idea that probiotics can modulate microbiome-gut-brain axis and influence brain function. Despite significant difference occurs between the human and mouse microbiomes, the evidence from experimental models suggest that changes of gut microbiota composition may affect molecular pathways involved in the onset and progression of anxiety- and depression-related behaviors **Figure 1**.

## Dysbiosis in depression and anxiety disorders: Evidences from human studies

In recent years, some studies were conducted to investigate how the intestinal microbiota play a role in patients with anxiety and mood disorders. In particular, several data from human studies shown that fecal microbiota often has some variability between patients and healthy controls, considering microbial diversity and taxonomic compositions. Furthermore, was reported that specific bacteria were associated with metabolic or inflammatory profiles and clinical characteristics (37).

Microbial diversity is a fundamental aspect in the study of fecal microbiota that is considered a marker of health, but the reproducibility of data is strongly limited by the interference of many environmental factors (38). To date, few studies reported data about microbial diversity in humans, most of these failed to demonstrate an association between lower microbial diversity and depressive disorders (39–41), while only one study reported higher α-diversity (i.e., the number of species detectable in a microbial ecosystem) of gut microbiota in major depressive disorder (MDD) patients compared to healthy subjects (42).

Taxonomic differences are described in several studies involving MDD patients, interesting differences have been reported for the main Phyla represented. Unfortunately, the findings from human studies are often conflicting, probably due to several confounding factors. For instance, several changes in microbial composition were reported in the Phylum of *Firmicutes*, but as previous discussed, findings were often contradictory. The relative abundance of this phylum appeared to be more represented in MDD according to some studies (41, 43), however this finding it was not confirmed by farther report (42). Moreover, more differences were reported at family level considering that *Lachnospiraceae* were found increased (40–42) or decreased (39) between available studies, likewise *Ruminococcaceae* had a fluctuating representation, higher (40, 41) or lower (42) among reports. Finally, the genus level showed the most remarkable changes that were described for *Faecalibacterium* (40, 42) and *Ruminococcus* (42), these genera were decreased in subjects with depressive disorders. Similarly, changes in microbial composition were described for other phyla such as *Bacteroidetes* (39, 41–43) and *Actinobacteria* (41, 42), although with sometimes conflicting results among the various studies. Most significative differences were observed at the genus level as a reduced representation of *Bifidobacterium* (44). Furthermore, correlation between clinical characteristics of patients and microbial signature was reported. Specifically, *Fusobacteria* and *Proteabacteria* appeared to be increased or reduced, respectively in active-MDD or recovering-MDD (42).

Above, we have briefly reported the complexity and the divergences between the evidences probably due to methodological differences and environmental variability among different studies. A recent systematic review showed that about 50 bacterial taxa exhibit differences between patients with MDD and controls (45). However, the authors failed to demonstrate the prevalence of a specific bacterial taxa in the development of depression.

In the near future, meta-proteomics studies should add further elements in the understanding the association between microbiota and the development of depression. A pioneering study by *Chen* and colleagues investigated the metabolomic profile in patients with MDD and it reported several significant differences in the pathway of bacterial proteins that were mainly involved in glucose metabolism and amino acid metabolism (46).

Interesting alterations of the fecal microbiota have also been identified in patients suffering from anxiety disorder. In particular were found a reduction in microbial richness and diversity in patients with generalized anxiety disorder (GAD), associated with reduced short-chain fatty acid producing bacteria such as *Eubacterium rectale* and *Fecalibacterium*, and an increase in *Escherichia*, *Shigella*, *Fusobacterium* and *Ruminococcus* (47). More importantly, these changes were not reversed in remissive GAD. Conversely, another study failed to demonstrate any correlation between intestinal dysbiosis and anxiety in female subjects (48), confirming the variability between human studies.

## Potential for therapy

Gut microbiota represents a new frontier in psychiatry. For this reason, antibiotics, probiotics, prebiotics and FMT were investigated for the treatment of anxiety (49) and depression (50). Psychobiotics define these therapeutic tools (51), in particular the main evidences on the modulation of the gut microbiota in depression and anxiety disorders were reported in the next paragraphs.

### Antibiotics

Antibiotics are deep modulators of gut microbiota, and consequently they appear to change, in a positive or negative way, the nature of several gastrointestinal or extra-intestinal disorders (52). Therefore, in consideration of their known effect on behavior, they have been proposed as a therapeutic tool also in psychiatry (53). Potential and beneficial effects were described in individual with depression or anxiety related disorders.

For instance, Minocycline has been identified as a potential novel treatment for depression taking into consideration its potent anti-inflammatory and neuroprotective effects (54). In recent years, several clinical trials investigated the potential role of this drug in the scenario of depression; meta-analyses that included three RCTs reported preliminary evidence for a significant antidepressant effect of minocycline. The antidepressant effect size was found to be large (SMD − 0.78; 95%CI; 0.4−1.33; p=0.005) with moderate heterogeneity of the pooled sample. However, the small number of published RCTs and small sample sizes were significant limitations to draw definitive conclusions (55). Furthermore, the broad-spectrum antibiotic Cycloserine was investigated for the treatment of anxiety disorders. A meta-analysis that included 21 studies that involved 1047 individuals with several psychiatrics disorders (phobia, social anxiety disorder, panic disorder, obsessive-compulsive disorder and post-traumatic stress disorder) showed that Cycloserine was associated with a small augmentation effect on exposure-based therapy and suggested that this effect was not modulated by the concurrent use of antidepressants (56). However, antibiotics have also been associated with a negative effect on mood disorders. In particular, recurrent exposure to antibiotics such as penicillins (OR 1.23; 95% CI, 1.18-1.29) or quinolones (OR 1.25; 95% CI, 1.15-1.35) appeared to be associated with increased risk for depression and anxiety (57).

### Probiotics

Probiotics are defined as live microorganisms that, upon administration in adequate amounts, confer a health benefit on the host (58). To date, some studies report results on the use of probiotics in the treatment of mood disorders, albeit with some limitations as the heterogeneity of enrolled patients and the variety of the administered mixtures (59). *Miyaoka* and colleagues investigated the role of *Clostridium butyricum* (CBM588) as adjunctive therapy in patients with treatment-resistant MDD. In this study was reported a significant improvement in depression scale after 8 weeks of treatment, suggesting a potential therapeutic role for this probiotic strain in combination with antidepressant drugs (60). Another clinical trial reported that a probiotic mixture (*L. helveticus* R00052 and *B. longum* R0175) was able to ameliorate the beck depression inventory (BDI) in individuals with mild to moderate MDD compared to placebo (61). Farther, the administration of a mixture of *L. acidophilus*, *L. casei* and *B. bifidum* resulted in a significant reduction of BDI score (62). Sometimes MDD patients experienced gastrointestinal disorders and in particular IBS, in this context *Majeed* and colleagues reported significant improvement of depression and IBS symptoms in patients treated with *Bacillus Coagulans* MTCC 5856 (63). Promising results were also reported about stress and anxiety. Indeed, *Lactobacillus plantarum* DR7 appeared to be beneficial in reducing symptoms and psychological scores (64).

However, not all studies documented positive results, maybe due to probiotic strain, concurrent medications or other unexplored factors. For instance, *Romijn* and colleagues demonstrated that a probiotic mixture (*L. helveticus* R0052 and *B. longum* R0175) failed to improve depressive symptoms in individuals with low mood not currently taking psychotropic medications (65). Finally, another study clearly showed that the probiotic *B. Longum* NCCC3001 reduced depression but not anxiety scores and increased quality of life in patients with IBS (66). Furthermore, the effects were associated with changes in brain activation patterns demonstrating that this probiotic reduces limbic reactivity (66).

### Prebiotics

Prebiotics are selectively fermented compounds promoting changes in both composition and activity of intestinal microbiota that offer benefits to the host (67). Few studies investigated the role of prebiotics in mood disorders. *Smith* and colleagues failed to demonstrate a significant effect of a prebiotic mixture (oligofructose enriched inulin) on mood scores in a cohort of healthy adults. However, participants reported greater well-being after consumption of inulin (68). Similarly, despite beta-glucan derived from *Saccharomyces cerevisiae* improved mood in stressed subjects, no significant differences in depression scores were observed compared to placebo (69). Moreover, another clinical trial failed to demonstrate that prebiotic supplementation improved depressive symptoms. Indeed, administration of galacto-oligosaccharides for eight weeks did not significantly modify BDI score in MDD patients compared to placebo and its effect was lower than that of probiotic mixture (61). On the other hand, prebiotics supplementation appeared to be more efficacious on psychiatric symptoms in IBS patients. Short-chain fructo-oligosaccharides (scFOS) showed beneficial effects in a population with gastrointestinal symptoms. Specifically, scFOS supplementation for four weeks resulted in a significantly improvement of depression and anxiety scores, furthermore this effect was associated to changes in microbiota composition including increase of *Bifidobacteria* in feces (70). However, another prebiotic galacto-oligosaccharide mixture (B-GOS) not improved anxiety and depression scale in individuals with functional bowel disorders, albeit some beneficial effects were reported for gastrointestinal symptoms (71).

These conflicting data confirm the need for further studies to better establish the patient cohorts and compounds more efficacious for this type of intervention.

### Synbiotics

Synbiotics are defined as a synergic mixture of probiotics and prebiotics that promote beneficial effects on health, in particular prebiotics are involving in favoring the colonization of the gut by probiotics (72). A small number of clinical trials that investigated the role of synbiotics in mood disorders have been published. A first trial demonstrated that a symbiotic mixture (*Lactobacillus casaei*, *Lactobacillus acidofilus*, *Lactobacillus bulgarigus*, *Lactobacillus rhamnosus*, *Bifidobacterium breve*, *Bifidobacterium longum*, *Streptococus thermophiles*, and fructo-oligosaccharide) was able to decrease HAM-D score and to improve depressive symptoms in patients with moderate MDD (73).

Afterwards, another clinical trial demonstrated the greater efficacy of symbiotic formulations compared to probiotics mixture alone in the treatment of mood disorder. In the clinical trial designed by *Haghighat* and colleagues (74), patients were randomly assigned to receive synbiotics (prebiotics: fructo-oligosaccharides, galacto-oligosaccharides, and inulin; probiotics: *Lactobacillus acidophilus* T16, *Bifidobacterium bifidum* BIA-6, *Bifidobacterium lactis* BIA-7, and *Bifidobacterium longum* BIA-8) or probiotics (the same mixture of synbiotics without prebiotics) or placebo for twelve weeks **Table 1**.

**Table 1:** Results from clinical trials on modulation of gut microbiota in anxiety and depression.

Type of drug	Drug	Effects	References
Antibiotics	Minocycline	anti-inflammatory, neuroprotective, anti depressant	(54, 55)
	Cycloserine	improves effect of conventional therapy on several psychiatric disorders	(56)
	penicillins	increased risk for depression and anxiety	(57)
	quinolones	increased risk for depression and anxiety	(57)
Probiotics	*Clostridium butyricum* (CBM588)	improves effect of conventional therapy in depression	(60)
	*L. helveticus* R00052 and *B. longum* R0175	Amelioration of the BDI in MDD, contrasting results by another study that failed to improve depressive symptoms	(61, 65)
	*L. acidophilus*, *L. casei* and *B. bifidum*	Reduction of BDI score	(62)
	*Bacillus Coagulans* MTCC 5856	Amelioration of depression and IBS symptoms	(63)
	*Lactobacillus plantarum* DR7	Amelioration in symptoms and psychological scores	(64)
	*B. Longum* NCCC3001	Ameliorate depression, improves quality of life, but not anxiety in IBS	(66)
Prebiotics	oligofructose enriched inulin	No significant effects on healthy subjects	(68)
	inulin	Improve weel-being in healthy subjects	(68)
	beta-glucan (derived from *Saccharomyces cerevisiae*)	No effect on depression score	(69)
	galacto-oligosaccharides	No changes on anxiety and depression scale	(61, 71)
	scFOS	Improves depression and anxiety score in IBS, correlating with the increase of Bifidobacteria	(70)
Synbiotics	*Lactobacillus casaei, Lactobacillus acidofilus, Lactobacillus bulgarigus, Lactobacillus rhamnosus, Bifidobacterium breve, Bifidobacterium longum, Streptococus thermophiles*, and fructo-oligosaccharide	Improve depressive symptoms in MDD	(73)
	fructo-oligosaccharides, galacto-oligosaccharides, and inulin*; Lactobacillus acidophilus T16, Bifidobacterium bifidum BIA-6, Bifidobacterium lactis BIA-7, and Bifidobacterium longum BIA-8*	Symbiotic mixture is superior to probiotics alone in improving depression an anxiety simptoms	(74)

The table report the main results described in human studies, however describe as within the same pharmacological class there are promising even if sometimes conflicting results.

### Fecal microbiota transplantation

Fecal microbiota transplantation is the infusion of a fecal suspension derived from a healthy donor into the intestine of a recipient to restore the imbalanced gut microbiota (75). Some fascinating studies on animal models have supported the idea that the transfer of “good microbes” can represent a new tool in the treatment of depression and anxiety. For example, it has been demonstrated that the transfer of healthy microbiota in an animal model of alcohol-induced anxiety and depression reduced the clinical manifestation in the animal (76). On the other hand, it was reported the “transfer of depression” trough microbiota. Indeed, germ free mice who underwent to FMT derived from MDD patients resulted in depression-like behaviors compared with colonization by microbiota derived from healthy control individuals (40). Furthermore, another study confirmed that FMT from depressed patients to microbiota-deficient rats could induce behavioral and physiological features characteristic of depression in the recipient animals, including anhedonia and anxiety-like behaviors (77). Unfortunately, the evidence for the use of FMT in humans is still limited (78, 79). A small study on 17 patients with functional gastrointestinal disorders treated with FMT reported an improvement of depression and anxiety symptoms independently of gastrointestinal symptom changes (80). A further small clinical study demonstrates that FMT in patients with IBS-D is able to reduce levels of anxiety and depression, as well as gastroenterological symptoms, in particular was associated to the decreased abundance of Faecalibacterium, Eubacterium and Escherichia (81). Further studies are needed to validate the procedure and to identify microbiome more efficacious for FMT.

## Final remarks

The microbiota-gut–brain axis is an integrative system that involves metabolic, immunological and neuroendocrine signals, and alterations of these pathways play relevant roles in human neurological diseases. Extensive research has demonstrated that diet, drugs and stress influence both composition and function of gut microbiota, which in turn can modulate neurophysiology and behavior. Therefore, gut microbiota represents a key mechanism underlying the impact of environmental stimuli on brain function and identifying the biological pathways involved in the microbiota-gut-brain axis may be relevant to understand the pathophysiology of human mood disorders. Further, developing therapeutic strategies to modify the composition of gut microbiota may offer novel and personalized therapeutic tools (82). Indeed, several studies have reported that treatments able to modify the intestinal microbiota exerted a significant effect on the symptoms of anxiety disorders and depression in humans. More specifically, treatment with probiotics and synbiotics showed the best results in terms of symptom improvement, suggesting a potential role as adjunctive therapy. Unfortunately, the results about prebiotics alone are not satisfactory in the setting of mood disorders. Results from FMT studies in humans are fascinating but still too weak. Finally, the evidences from antibiotic studies are conflicting (83), because while some drugs such as minocycline and cycloserine have shown to have beneficial effects, other drugs of wide clinical use, as penicillins or quinolones, may increase the risk for depression and anxiety. In this review we have analyzed how some pharmacological approaches can modify the gut microbiota and promote a favorable effect on anxiety and depression. On the other hand, in recent years, “non-pharmacological” treatments are also being considered to regulate microbiota composition. It is known that diet plays a fundamental role in modulating the microbiota (84), this is true both in health and in disease. In particular, several evidences are emerging on how diet can play a role in the treatment of behavioral disorders (85). For instance, it has been shown that a diet rich in fat can favor the development or persistence of anxiety and depression, an effect sometimes reversible with probiotics (86). Furthermore, experimental models have shown how a supplementation diet with psychoactive metabolites, such as tryptophan, can have a protective role on the development of these mood disorders through the reduction of stress-induced gut barrier damage and inflammatory responses in the gut (87). Still reporting on non-pharmacological approaches, in the last year very interesting results have emerged from studies evaluating the role of cognitive behavioral therapy (CBT) in modifying the microbiota. For instance, a small study demonstrate that mindfulness CBT promote changes in gut microbiota of subjects affected by anxiety, in particular the individuals who responded better by reducing anxiety modified the microbiota making it more similar to healthy subjects and interestingly they increased the metabolism of tryptophan (88). The interpretation of these results opens up new frontiers on the modulation of the gut-brain axis, in fact it appears possible to modulate it in both directions (gut-brain and brain-gut) to obtain modifications for therapeutics.

In conclusion, drugs and non-pharmacological approaches regulating the composition of intestinal microbiota represent promising beneficial strategies against anxiety and depression. The study of the crosstalk between microbiota and brain can improve knowledge about the development of mood disorders and help to identify new therapeutic tools for the personalized medicine.

## Author contributions

SB, SF, and AG contributed to conception and design of the study. SB and SF wrote the first draft of the manuscript. All authors contributed to manuscript revision, read, and approved the submitted version

## Funding

This paper received funding from Fondazione Roma.

## Acknowledgments

The authors thank Fondazione Roma for the continuous non-conditioning support.

## Conflict of interest

The authors declare that the research was conducted in the absence of any commercial or financial relationships that could be construed as a potential conflict of interest.

## Publisher’s note

All claims expressed in this article are solely those of the authors and do not necessarily represent those of their affiliated organizations, or those of the publisher, the editors and the reviewers. Any product that may be evaluated in this article, or claim that may be made by its manufacturer, is not guaranteed or endorsed by the publisher.

## Abbreviations

MDD, Major depressive disorders; GAD, Generalized anxiety disorder; IBS, Irritable bowel syndrome; SCFA, short chain fatty acid; BDI, Beck Depression Inventory; IBS, irritable bowel syndrome; HAM-D, Hamilton rating scale for depression; HADS, Hospital Anxiety and Depression Scale; CBT, cognitive behavioral therapy.
